# Supplementary material for: Longitudinal analysis of drift in the circulating human antibody repertoire over four years
Source: bioRxiv. 2025 Mar 25:2025.03.20.644446. Preprint. [Version 1] doi: 10.1101/2025.03.20.644446 (PMC11974760; doi:10.1101/2025.03.20.644446)
Supplement: Supplement 1 [file media-1.pdf]

Supplemental Information for

Longitudinal analysis of drift in the circulating human antibody repertoire over four years

Authors: Collin Joyce[1,2,3#], Benjamin Nemoz[1,2,4#], Raiza Bastidas[1,2,3], Bryan Briney[1,2,4,5,**] and Dennis R. Burton [1,2,3,6,*] **Affiliations:**

[1]Department of Immunology and Microbiology, The Scripps Research Institute, La Jolla, CA, USA

[2]Consortium for HIV/AIDS Vaccine Development (CHAVD), The Scripps Research Institute, La Jolla, CA, USA

[3]IAVI Neutralizing Antibody Center, The Scripps Research Institute, La Jolla, CA, USA

[4]Center for Viral Systems Biology, The Scripps Research Institute, La Jolla, CA, USA

[5]Multi-Omics Vaccine Evaluation (MOVE) Consortium, The Scripps Research Institute, La Jolla, CA, USA

[6]Ragon Institute of MGH, MIT and Harvard, Cambridge, MA, USA

# Co-first authors

**Correspondence: briney@scripps.edu

*Correspondence: burton@scripps.edu

This PDF file includes:

Figures S1 to S4 and Table S1

Supplemental Figure 1. V/J frequency correlations of technical and biological replicates. For each sample, the frequency of V and J combinations was compared for technical replicates (left panels) or biological replicates (right panels). The coefficient of determination (r2) is shown for each plot.

Figure S1

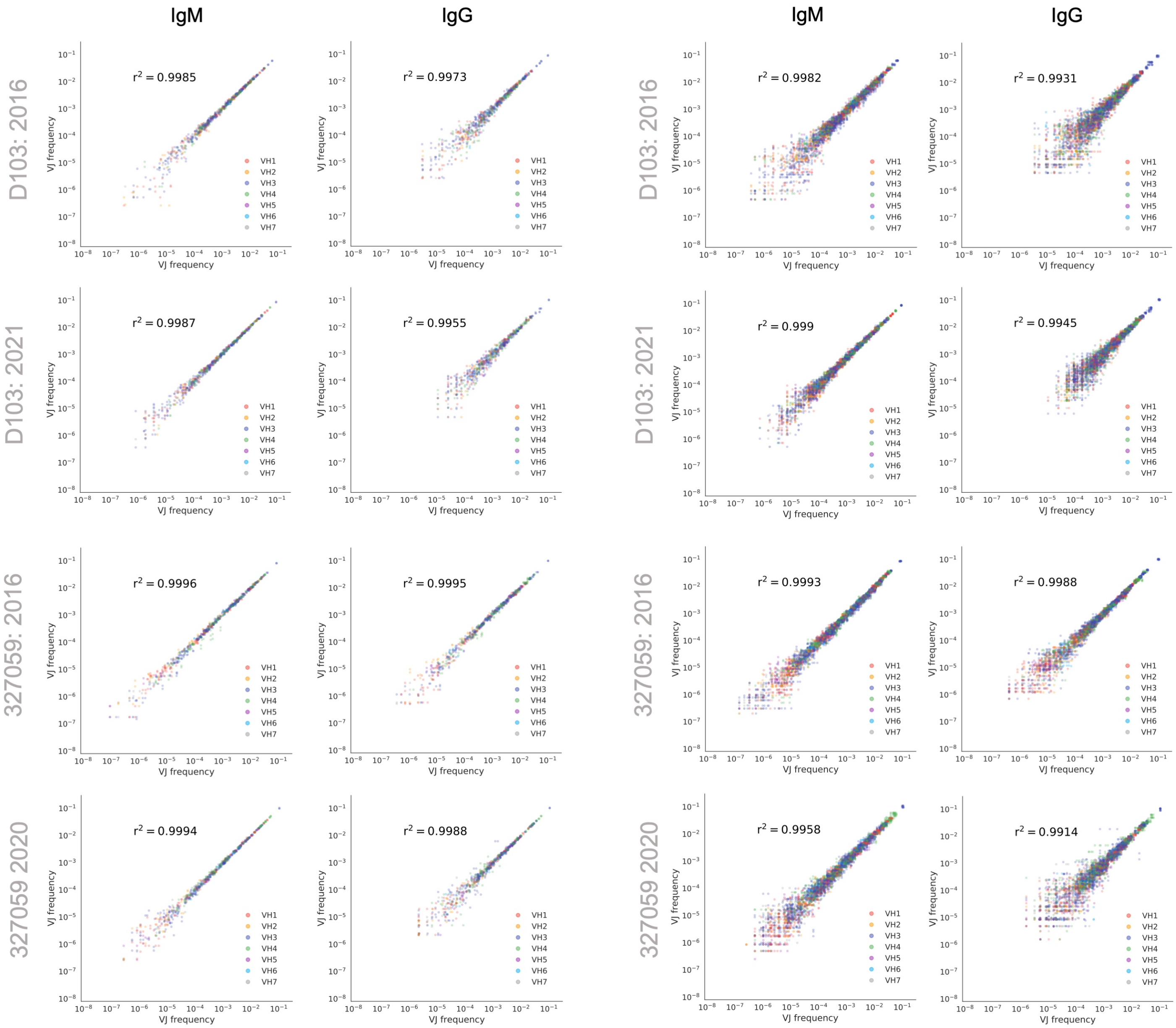
IgM
IgG
IgM
IgG
D103: 2016
D103: 2021
327059: 2016
327059 2020
$r^2 = 0.9985$
$r^2 = 0.9973$
$r^2 = 0.9982$
$r^2 = 0.9931$
$r^2 = 0.9987$
$r^2 = 0.9955$
$r^2 = 0.999$
$r^2 = 0.9945$
$r^2 = 0.9996$
$r^2 = 0.9995$
$r^2 = 0.9993$
$r^2 = 0.9988$
$r^2 = 0.9994$
$r^2 = 0.9988$
$r^2 = 0.9958$
$r^2 = 0.9914$
VJ frequency
VJ frequency
VH1
VH2
VH3
VH4
VH5
VH6
VH7

Supplemental Figure 2. V/J frequency correlations between timepoints. For each subject, the frequency of V and J combinations was compared between timepoints for all isotypes (left), IgM sequence (center) and IgG sequences (right). The coefficient of determination (r2) is shown for each plot.

Figure S2

Inter-year comparisons

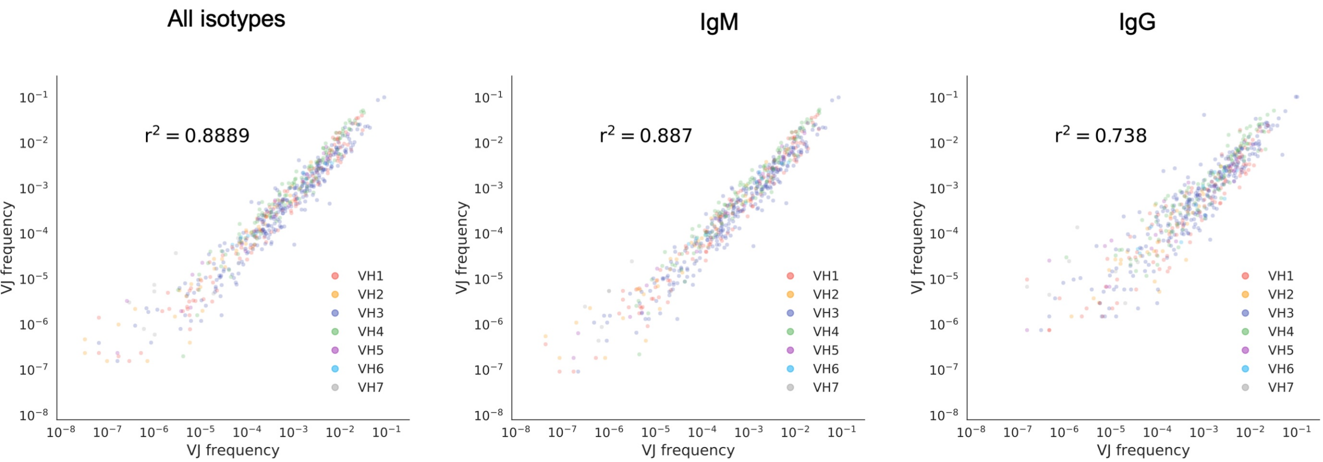

Supplemental Figure 3. Percent shared clonotypes (solid line) and sequences (dotted line) verses sample depth. Lines indicate mean frequency of 10 bootstrap samplings.

Figure S3

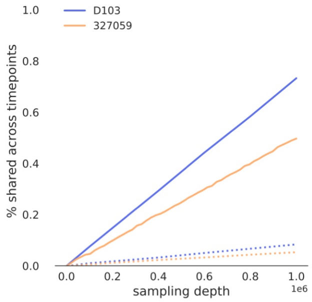

D103
327059
% shared across timepoints
1.0
0.8
0.6
0.4
0.2
0.0
0.0
0.2
0.4
0.6
0.8
1.0
1e6
sampling depth

Supplemental Figure 4. Public clonotype properties for each timepoint. Top panel shows sequence logos of CDRH3s of length 13 amino acids encoded by shared (top) and unshared (bottom) clonotypes between donors at each timepoint. Bottom panel shows distribution of CDRH3 length for shared and unshared public clonotypes between donors at each timepoint.

Figure S4

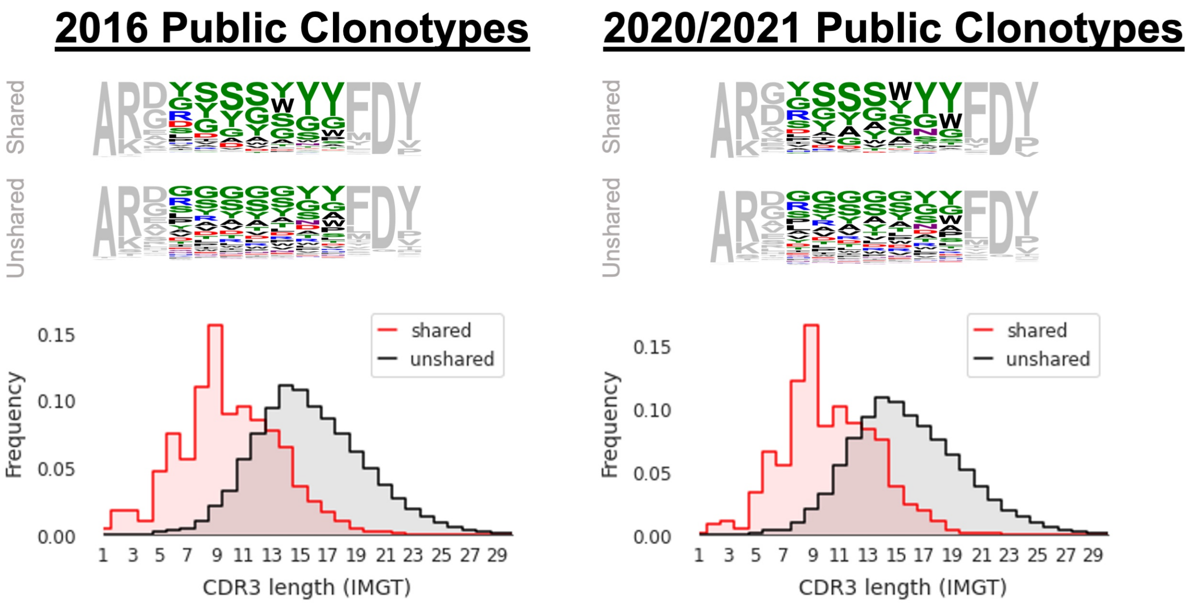
2016 Public Clonotypes
2020/2021 Public Clonotypes
Shared
Unshared
shared
unshared
Frequency
0.00
0.05
0.10
0.15
1 3 5 7 9 11 13 15 17 19 21 23 25 27 29
CDR3 length (IMGT)

Subject	Timepoint #1	Timepoint #2	Age at Timepoint #1	Age at Timepoint #2	Raw reads	Consensus sequences	Clonotypes
327059	11/22/16	12/2/20	26	30	801,514,273	44,035,482	12,900,996
D103	11/17/16	4/1/21	25	30	473,638,628	16,862,355	4,453,581

Supplemental Table 1. Per-subject timepoint information and sequencing statistics.
